# The application of methylation specific electrophoresis (MSE) to DNA methylation analysis of the 5' CpG island of mucin in cancer cells

**DOI:** 10.1186/1471-2407-12-67

**Published:** 2012-02-14

**Authors:** Seiya Yokoyama, Sho Kitamoto, Norishige Yamada, Izumi Houjou, Tamotsu Sugai, Shin-ichi Nakamura, Yoshifumi Arisaka, Kyoichi Takaori, Michiyo Higashi, Suguru Yonezawa

**Affiliations:** 1 Department of Human Pathology, Field of Oncology, Kagoshima University Graduate School of Medical and Dental Sciences, 8-35-1 Sakuragaoka, Kagoshima 890-8544, Japan; 2 Division of Pathology, Central Clinical Laboratory, School of Medicine, Iwate Medical University, Morioka, Japan; 3 DPR Co., LTD. 4-10-53, Mitake, Morioka 020-0122, Japan; 4 Second Department of Internal Medicine, Osaka Medical College, 2-7 Daigaku-machi, Takatsuki, Osaka 569-8686, Japan; 5 Division of Hepato-Biliary-Pancreatic Surgery and Transplantation, Department of Surgery, Kyoto University Graduate School of Medicine, 54 Kawara-cho, Shogoin, Sakyo-ku, Kyoto 606-8507, Japan

**Keywords:** DNA methylation pattern, Epigenetics, Mucin, Colonic crypt, Pancreatic juice, Cancer

## Abstract

**Background:**

Methylation of CpG sites in genomic DNA plays an important role in gene regulation and especially in gene silencing. We have reported mechanisms of epigenetic regulation for expression of mucins, which are markers of malignancy potential and early detection of human neoplasms. Epigenetic changes in promoter regions appear to be the first step in expression of mucins. Thus, detection of promoter methylation status is important for early diagnosis of cancer, monitoring of tumor behavior, and evaluating the response of tumors to targeted therapy. However, conventional analytical methods for DNA methylation require a large amount of DNA and have low sensitivity.

**Methods:**

Here, we report a modified version of the bisulfite-DGGE (denaturing gradient gel electrophoresis) using a nested PCR approach. We designated this method as methylation specific electrophoresis (MSE). The MSE method is comprised of the following steps: (a) bisulfite treatment of genomic DNA, (b) amplification of the target DNA by a nested PCR approach and (c) applying to DGGE. To examine whether the MSE method is able to analyze DNA methylation of mucin genes in various samples, we apply it to DNA obtained from state cell lines, ethanol-fixed colonic crypts and human pancreatic juices.

**Result:**

The MSE method greatly decreases the amount of input DNA. The lower detection limit for distinguishing different methylation status is < 0.1% and the detectable minimum amount of DNA is 20 pg, which can be obtained from only a few cells. We also show that MSE can be used for analysis of challenging samples such as human isolated colonic crypts or human pancreatic juices, from which only a small amount of DNA can be extracted.

**Conclusions:**

The MSE method can provide a qualitative information of methylated sequence profile. The MSE method allows sensitive and specific analysis of the DNA methylation pattern of almost any block of multiple CpG sites. The MSE method can be applied to analysis of DNA methylation status in many different clinical samples, and this may facilitate identification of new risk markers.

## Background

The gene expression profile of a cancer specimen is a valuable source of biological information that has potential clinical utility [1]. However, RNA or DNA of a sufficient quality and quantity for analysis may not be recoverable from clinical samples. Methylation of CpG sites in genomic DNA plays an important role in gene regulation, especially in gene silencing, and the promoter region of a transcribed gene is generally hypomethylated. Mechanisms of epigenetic regulation for expression of various mucins (MUC1, MUC2, MUC3A, MUC4, MUC5AC and MUC17) are reported [2-10]. Furthermore, abnormal epigenetic changes appear to be early "seeds of methylation" that precede detection of genetic mutations [11,12]. Thus, a method for detecting alteration of methylation status could be a valuable tool for early diagnosis of cancer, monitoring of tumor behavior, and assessing the response of tumors to targeted therapy.

**Figure 1:**
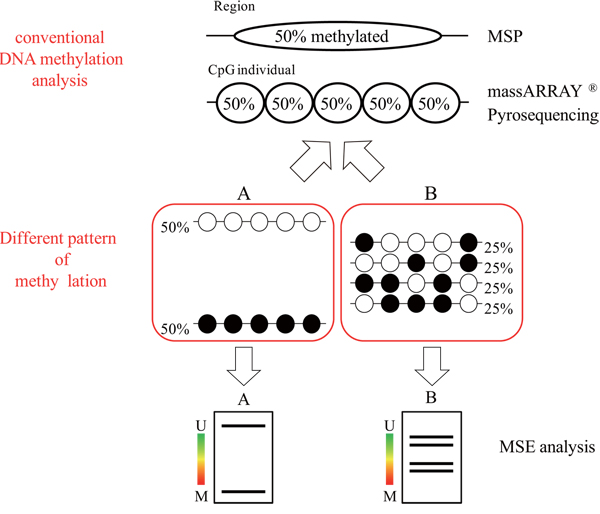
**Problems with current analysis methods**. For crude samples, MSP may indicate that "a region was 50% methylated" while massARRAY indicates that "all CpG sites are 50% methylated". The methylation pattern may be A (extreme pattern) or B (variable pattern). ○, unmethylated CpG site; ●, methylated CpG site.

Several methods for analyzing DNA methylation have been developed, each of which ideally requires a large amount of high-quality DNA, such as that obtained from cultured cells or fresh tissue samples. However, DNA recovered from clinical samples such as urine sediment, saliva, sputum, bronchial washing fluid, bile or pancreatic juice is often limited in quantity or degraded. Development of DNA methylation as a marker involves examination of the correlation between the extent of DNA methylation and pathological findings, and such studies have been performed using human fluid samples such as pancreatic juice, bile juice and blood [13-18]. These studies have often used the methylation specific PCR (MSP), methylight or massARRAY analysis. However, as shown in Figure 1, in analysis of crude samples, MSP and methylight may indicate that "a region was 50% methylated" while massARRAY indicates that "all CpG sites were 50% methylated". Thus, the methylation pattern might be A (extreme pattern) or B (variable pattern). These 2 patterns are very different, but current methods cannot distinguish between A and B. This is a pitfall for accurate diagnosis in clinical application of DNA methylation analysis. Thus, a DNA methylation pattern with information on content is very important for evaluation of the DNA methylation status.

To resolve this issue, we developed a modified version of the bisulfite-denaturing gradient gel electrophoresis (DGGE) [19] which used a nested PCR approach to increase the sensitivity of detection of DNA methylation pattern, since the DGGE can detect disparity of single mutation in the same size PCR product. We designated this method as methylation specific electrophoresis (MSE). The MSE method allows sensitive and specific analysis of the DNA methylation pattern of almost any block of multiple CpG sites. We applied this method to analyze the DNA methylation patterns of promoter regions of various mucins in 12 cancer cell lines. In addition, we examined the DNA methylation patterns of the promoter region of MUC1 in samples of human isolated colonic crypts, and also examined human pancreatic juices collected from the patients with pancreatic ductal adenocarcinoma (PDAC) having a very poor prognosis and from the patients with intraductal papillary mucinous neoplasm (IPMN) having a favorable prognosis.

## Methods

### Cell lines

Human pancreatic carcinoma cell lines HPAF II, BxPC3, PANC1; human breast cancer cell lines MCF-7, T-47D, MDA-MB-453; and human colon adenocarcinoma cell lines Caco2 and LS174T were obtained from American Type Culture Collection. HPAF II, MCF-7, LS174T, and Caco2 cells were cultured in Eagle's MEM (Sigma-Aldrich), PANC1 cells were cultured in DMEM (Sigma-Aldrich), BxPC3 and T-47D cells were cultured in RPMI 1640 (Sigma-Aldrich), and MDA-MB-453 cells were cultured in Leibovitz's L-15 medium (Invitrogen, Carlsbad, CA, USA). All media were supplemented with 10% fetal bovine serum (Invitrogen, Carlsbad, CA, USA), 100 U/ml penicillin (Sigma-Aldrich) and 100 μg/ml streptomycin (Sigma-Aldrich).

### Isolation of human colonic crypts

Fresh normal and tumor tissue samples were obtained from colorectal carcinoma specimens that were obtained surgically at Iwate Medical College Hospital. Normal colonic mucosa was taken from an area distant from the tumor. The tumor samples were obtained from the tumor-rich area of carcinomas. Crypt isolation from the normal and neoplastic mucosa was performed as previously reported [20-22]. Briefly, fresh normal mucosae and tumors were minced with a razor into minute pieces and then incubated at 37°C for 30 minutes in calcium- and magnesium-free Hanks' balanced salt solution (CMF) containing 30 mmol/L ethylene-diaminetetraacetic acid (EDTA). Following this procedure, the tissue was stirred in CMF for 30-40 minutes. Normal and neoplastic glands were separated from the lamina propria mucosa or fibrous stroma. The isolated crypts were immediately fixed in 70% ethanol and stored at 4°C until used for DNA and RNA extraction, and were also embedded in paraffin for immunohistochemical staining. All studies using human materials in this article were approved by the ethical committees of Kagoshima University hospital and other hospitals.

### Pancreatic juice

After completion of endoscopic retrograde pancreatography, pancreatic juice was collected using endoscopic nasopancreatic drainage (ENPD) or pancreatic stenting in 2 patients with PDAC and 5 patients with IPMN at Osaka Medical College Hospital [23]. Collection of the samples was approved by the ethical committee of the hospital and informed consent was obtained from each patient. All studies using human materials in this article were approved by the ethical committees of Kagoshima University hospital and other hospitals.

### Extraction and quantification of mRNA

#### Extraction of RNA

Total RNA was extracted from the cell lines, human colonic crypts and pancreatic juices using a RNeasy Mini kit (QIAGEN, Chuo-ku, Tokyo, Japan). Total RNA of 1 μg was then reverse transcribed with a High Capacity RNA-to-cDNA Kit (Applied Biosystems, Foster City, CA, USA).

#### Real time PCR assay

For quantification of MUC1 mRNA in the ethanol-fixed isolated crypts from human colonic normal mucosa and carcinoma lesions, the real time PCR assay was performed as described previously [7]. The primers and probes were designed and synthesized by Applied Biosystems. The product number of the Target Assay Mix used for MUC1 was Hs00410317. Human glyceraldehyde-3-phosphate dehydrogenase (GAPDH; product number 4310884E) was used to calibrate the original concentration of mRNA; i.e., the concentration of mRNA in the cell was defined as the ratio of target mRNA copies versus GAPDH mRNA copies. In this analysis, data from three separate experiments were averaged.

#### RT-PCR

For confirmation of gene expression level in cell lines, semiquantitative RT-PCR were performed using a Fast Cycling PCR kit (QIAGEN, Japan). The RT-PCR products were subjected to electrophoresis on 1% agarose gel. Differentially expressed genes were detected using the primer pairs (shown in Additional file 1: Table S1).

### Extraction of DNA and bisulfite modification

DNA from cell lines, ethanol-fixed human crypt sections, and pancreatic juice samples was extracted using a DNeasy Tissue System (QIAGEN, Chuo-ku, Tokyo, Japan). Bisulfite modification of the genomic DNA was carried out using an Epitect Bisulfite Kit (QIAGEN, Chuo-ku, Tokyo, Japan).

### Preparation of the samples for MSE method

#### Primer design

The target sequence containing CpG sites important for expression in respective mucin promoter regions were determined using a massARRAY analysis that has been published previously [2-10]. All the PCR primers were designed to avoid CpG site. The primers are bisulfite genomic sequencing type (Table 1). In addition, the target PCR primers were designed to be optimal sample fragment sizes (100 bp to 700 bp) for DGGE (Table 1B).

**Table 1:** Synthetic oligonucleotides used in MSE and conventional bisulfite-DGGE

Primer name		sequence	Tm
A. The nested PCR primer set			
MUC1	Forward:	5'-AAAGGGGGAGGTTAGTTGGA-3'	63
	Reverse:	5'-TACCCCTCACCTATAAACAC-3'	
MUC2	Forward:	5'-TTTGGGGTTAGGTTTGGAAG-3'	59
	Reverse:	5'-ACCTTCTTCAAAATAAAACAACC-3'	
MUC3A	Forward:	5'-TTGAGGGATAGAAGGGGTTTG-3'	64
	Reverse:	5'-AACCCCAACAACTACATAAACCC-3'	
MUC4	Forward:	5'-AGAGTAAGGGGTGTATGGGTG-3'	60
	Reverse:	5'-ACTCCACTACCCAACAACTAC-3'	
MUC5AC	Forward:	5'-AAAGTTTTGGGTGTGTGGAG-3'	62
	Reverse:	5'-ATCAATATCCAACCCCCAAC-3'	
MUC17	Forward:	5'-ATAAAGGGGGTGTTTTTGTTAGG-3'	62
	Reverse:	5'-AAACAAACAAAACAAACTAACCCC-3'	
B. The target PCR primer set			
MUC1	Forward:	5'-[GC CLAMP]AAGAGGTAGGAGGTAGGGGA-3'	53
	Reverse:	5'-AAAACAAAACAAATTCAAAC-3'	
MUC2	Forward:	5'-[GC CLAMP]TTTTAGAGTTTGGGTTTTAG-3'	51
	Reverse:	5'-TAACCTAAATACCAACACAC-3'	
MUC3A	Forward:	5'-[GC CLAMP]TTTTAGGTAGTTTTATGTGG-3'	52
	Reverse:	5'-AACAAAAAACTAAAACAAAAC-3'	
MUC4	Forward:	5'-[GC CLAMP] AGGAGAGAAAAGGGTGATTAG -3'	57
	Reverse:	5'-ACTCCACTACCCAACAACTAC-3'	
MUC5AC	Forward:	5'-[GC CLAMP]TTTATGTTTAGGGGTTTTGG-3'	62
	Reverse:	5'-ACCAACTAACCACCCAAACC-3'	
MUC17	Forward:	5'-[GC CLAMP]ATTTTTATGTTTATGGGTTG-3'	53
	Reverse:	5'-ATAATCCCTAACCTTAACATC-3'	

*[GC CLAMP]:5'-CGCCCGCCGCGCGCGGCGGGCGGGGCGGGGGCACGGGGGG-3'

#### Preparation of the samples

In the MSE method, a nested-PCR approach was used. In the first round of PCR, bisulfite treated DNA was amplified using the nested primer sets (Table 1A). The cycling conditions consisted of an initial denaturation step at 95°C for 5 min, then 40 cycles of 96°C for 5 s, Tm°C for 5 s, and 68°C for 3 s (the value of Tm shown in Table1). The information of each PCR product size and primer location at this initial PCR reaction is shown in Table 2A. For the second round of PCR, this product was diluted 1:50 in water, and 2 μl of the dilution were amplified using the target primer sets (Table 1B). The PCR parameters were as above, except that the annealing temperatures for the MUC1, MUC2, MUC3A, MUC4, MUC5AC and MUC17 reactions were 53, 51, 52, 57, 62 and 53°C respectively. The information of each PCR product size and primer location at the second PCR reaction is shown in Table 2B. All steps of PCR were performed using a Fast Cycling PCR kit (QIAGEN, Japan).

**Table 2:** Information of PCR product size and primer location

Primer name		location*	Product size**
A. The nested PCR primer set			
MUC1	Forward:	-219 bp to -200 bp	375 bp
	Reverse:	+156 bp to +137 bp	
MUC2	Forward:	-460 bp to -441 bp	261 bp
	Reverse:	-200 bp to -219 bp	
MUC3A	Forward:	-478 bp to -418 bp	495 bp
	Reverse:	+17 bp to -6 bp	
MUC4	Forward:	-249 bp to -228 bp	239 bp
	Reverse:	-30 bp to -11 bp	
MUC5AC	Forward:	-3960 bp to -3941 bp	450 bp
	Reverse:	-3511 bp to -3530 bp	
MUC17	Forward:	-383 bp to -361 bp	586 bp
	Reverse:	+203 bp to +180 bp	
B. The target PCR primer set			
MUC1	Forward:	-124 bp to -15 bp	206 bp
	Reverse:	+42 bp to +23 bp	
MUC2	Forward:	-437 bp to -418 bp	256 bp
	Reverse:	-222 bp to -241 bp	
MUC3A	Forward:	-321 bp to -302 bp	361 bp
	Reverse:	-10 bp to -31 bp	
MUC4	Forward:	-194 bp to -173 bp	156 bp
	Reverse:	-59 bp to -38 bp	
MUC5AC	Forward:	-3842 bp to -3823 bp	225 bp
	Reverse:	-3658 bp to -3677 bp	
MUC17	Forward:	-200 bp to -181 bp	312 bp
	Reverse:	+72 bp to +53 bp	

* location*: relative to transcription start site. Product size

**: GC clamp added size

### Denaturing gradient gel electrophoresis (DGGE)

After the PCR, DGGE was performed as described by Schäfer and Muyzer [24] using the D-Code system (Bio-Rad Laboratories, Hercules, CA, USA). Electrophoresis was performed with 1-mm thick 10% polyacrylamide gels (ratio of acrylamide to bisacrylamide, 40:1) submerged in 1Χ TAE buffer (40 mM Tris, 40 mM acetic acid, 1 mM EDTA, pH 7.5) at a constant temperature of 60°C. PCR products in amounts ranging from 5 μl were applied to the individual lanes on the gel. The electrophoresis conditions for the target gene fragment were 14 h at 70 V in a linear denaturant gradient (MUC1, 30%-40%; MUC2, 25%-35%; MUC3A, 25%-35%; MUC4, 25%-45%; MUC5AC, 30%-40%; MUC17, 35%-45%). After electrophoresis, the gels were incubated for 30 min in Milli-Q water containing GelRed (dilution 1:10000) (Biotium, Hayward, CA, USA) and photographed using AE-6905 N (ATTO). The emission intensity of the band was measured using Image J (NIH).

### Conventional bisulfite-DGGE for comparison

In the conventional bisulfite-DGGE method, the bisulfite treated DNA was amplified using the target primer sets (Table 1B). PCR parameters were as above, except that the annealing temperatures for the MUC1, MUC2, MUC3A, MUC4, MUC5AC and MUC17 reactions were 53, 51, 52, 57, 62 and 53°C respectively. Information on each PCR product size and each primer location is shown in Table 2B. All steps of PCR were performed using a Fast Cycling PCR kit (QIAGEN, Japan). After the PCR, these products were applied to DGGE.

### Immunohistochemical staining

MUC1 protein expression was assessed by immunohistochemistry using an anti-MUC1 monoclonal antibody (MAb) clone 014E (Mab MUC1-014E), which was developed by Yonezawa et al. [25], in ethanol-fixed paraffin-embedded 4-μm sections of the isolated human normal and neoplastic crypts. Immunohistochemistry was performed by the immunoperoxidase method as follows. Antigen retrieval was performed using CC1 antigen retrieval buffer (Ventana Medical Systems, Tucson, AZ, USA) for all sections. Following incubation with MAb MUC1-014E (diluted 1:5) in phosphate-buffered saline (PBS, pH 7.4) with 0.1% bovine serum albumin, sections were stained on a Benchmark XT automated slide stainer using a diaminobenzidine detection kit (Ventana Medical Systems).

## Results and discussion

### MSE approach for MUC1 promoter region

We examined the characteristics of the MSE approach for MUC1 using cell lines in which the DNA methylation status of the MUC1 promoter region has been precisely defined [7]. First, we made controls of fully methylated and unmethylated DNA. The completely fully methylated control DNA was prepared by the treatment using methyltransferase (*Sss *I) on DNA from Caco2 which has the highest methylation status in the cell lines examined in the previous MassARRAY analysis (Additional file 2: Table S2) [7]. Similarly, the fully unmethylated control DNA was prepared by PCR for the MUC1 promoter region on DNA from T-47D which has the lowest methylation status in the cell lines examined in the previous MassARRAY analysis (Additional file 2: Table S2) [7]. The bisulfite-sequencing analyses for the fully methylated and unmethylated controls showed clearly defined methylated and unmethylated sequence (Additional file 3: Table S3). The MSE analyses for the fully methylated and unmethylated controls showed clearly defined methylated and unmethylated bands (Additional file 4: Figure S1). This electrophoretic migration distance depends on mutation (cytosine vs. thymine) in the target sequence, but not on the PCR product size. Thus, the "methylated sequence" carrying many cytosines shows higher mobility than the "un-methylated sequence" carrying many thymines which are converted from cytosines by the bisulfite treatment. For this reason, the more a band migrates, the higher the band is methylated. In addition, the multiple bands indicate a mix of various patterns of methylated CpG sequences. This method provides information on the degree of methylation status by the migration distance of bands and the variation of methylated sequences by the numbers of bands, although it does not provides the individual CpG methylation ratio.

### Sensitivity of MSE in MUC1 promoter region analysis

**Figure 2:**
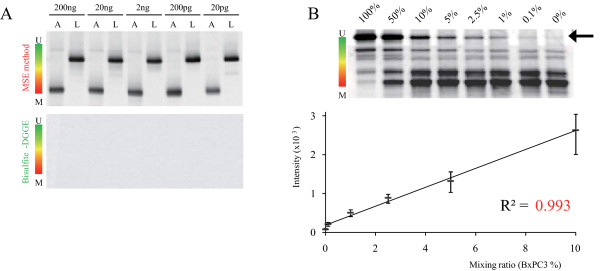
**(A) Analysis of the methylation status of A427 (A) and LS174T (L) by MSE and conventional bisulfite-DGGE**. (B) Determination of the resolution of MSE detection. A mixture of a high methylation cell line (Panc1) and a low methylation cell line (BxPC3) was analyzed. The number shows the BxPC3 contamination ratio. The upper band (arrow) shows the digitalized emission intensity using Image J. On the graph, the Y-axis shows the intensity of the upper band and the X-axis shows the BxPC3 contamination ratio. Regression equation: Y=243.26X+187.25; correlation co-efficient: R^2 ^= 0.993. Data were averaged from 3 separate experiments.

To examine the minimum volume for detection, we evaluated 5 concentrations (from 200 ng to 20 pg) of DNA extracted from the A427 and LS174T. Five concentration samples were prepared by a ten-fold serial dilution of initial bisulfite treated DNA sample (200 ng/μl). MSE was able to analyze 20 pg DNA of the bisulfite-treated DNA (Figure 2A, upper panel), whereas analysis with conventional bisulfite-DGGE was unsuccessful at under 200 ng DNA (Figure 2A, lower panel). Thus, MSE enhanced the overall sensitivity for detection of methylated MUC1 promoter DNA by at least 10,000-fold relative to conventional bisulfite-DGGE. In addition, we evaluated 5 concentrations of bisulfite treated DNA recovered from human pancreatic juice. Five concentration samples were prepared by a ten-fold serial dilution of initial bisulfite treated DNA sample (20 ng/μl). MSE was able to analyze 200 pg DNA of bisulfite-treated DNA (Additional file 5: Figure S2).

### Resolution of MSE in MUC1 promoter region analysis

Next, we analyzed DNA derived from BxPC3 (tendency for lower methylation of the MUC1 promoter region) and Panc1 (tendency for higher methylation of the MUC1 promoter region) (Figure 2B, upper panel). These 2 cell lines has heterogeneity of methylated CpG sequence pattern [7]. Thus, BxPC3 shows various bands which are inclined toward the hypomethylated side. Similarly, Panc1 shows various bands which are inclined toward the hypermethylated side. Aggerholm et al demonstrated a correlation exists between the band migration and the number of methylated sites in a denaturing gradient gel [19]. In addition, we tested MSE on a mixture of DNA derived from BxPC3 and Panc1 (Figure 2B, upper panel). We found that the emission intensity of the band for the hypo-methylated MUC1 promoter was directly proportional to the contamination ratio of hypo-methylated cells (R^2 ^= 0.993; Figure 2B, lower panel). MSE can accurately detect a methylation target at a level of 0.1%. These results indicate that MSE can detect 3 abnormal cells (un-methylated MUC1 promoter) in 3000 normal (methylated MUC1 promoter) cells. We have shown that MSE is not only highly specific and sensitive, but that it also can rapidly detect biologically relevant information in patient samples. In contrast, Li et al. suggested that an optimized MSP assay was unable to detect methylated templates when the fraction of methylated fragments was < 6.2% in parallel assays [26]. MSE provides significant advantages over the conventional bisulfite-DGGE since MSE requires only 200 pg of DNA of low quality, making it suitable for small biopsies, ethanol-fixed tissue, and clinical fluid samples.

### Application of MSE for promoter regions of various mucins

**Figure 3:**
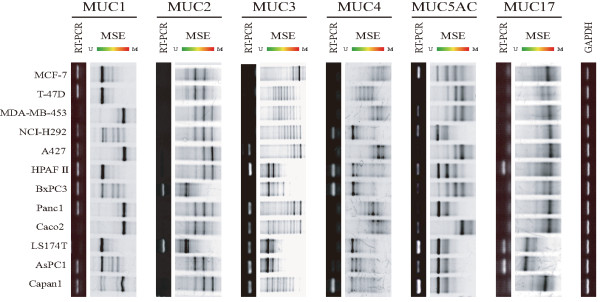
**MSE analysis of DNA methylation status of MUC1, MUC2, MUC3, MUC4, MUC5AC and MUC17 promoter regions in 12 cell lines**. The results of each mRNA analysis using RT-PCR are shown in the upper row of MSE results. The expression level of GAPDH mRNA in each cell line was used as a control in the RT-PCR analysis. The methylation pattern of each gene was consistent with the results of massARRAY analysis [2,4,6-10].

To show that the MSE method can be used to analyze promoter regions of various mucins (MUC1, MUC2, MUC3A, MUC4, MUC5AC and MUC17), we analyzed the DNA methylation status in promoter regions that play key roles in expression of mucins in 12 cancer cell lines. Expression of mRNA was detected by semiquantitative RT-PCR analysis, with GAPDH mRNA used as a control. The expression level of mRNA shows a high propensity for regulation by DNA methylation [2,4,6-10] (Figure 3). The results of MSE analysis in each cell line (Figure 3) were consistent with DNA methylation patterns obtained by massARRAY analysis [2,4,7-10].

### Application of MSE for human samples

**Figure 4:**
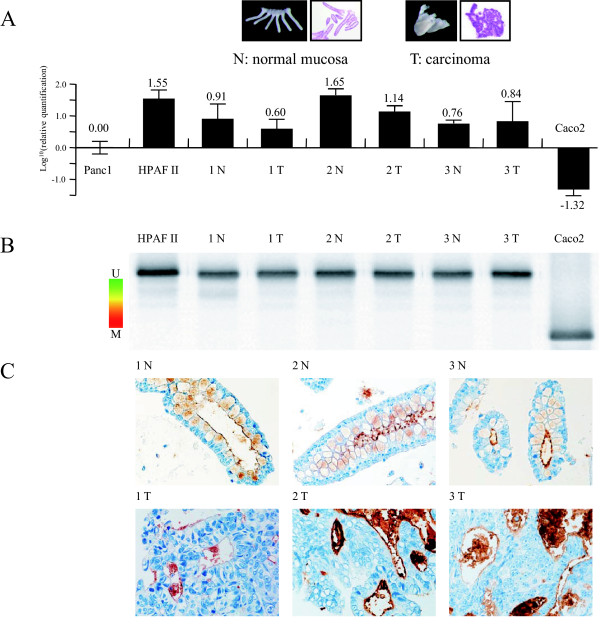
**Epigenetic analysis of MUC1 expression in human colonic normal and neoplastic crypts**. (A) Expression of *MUC1 *mRNA examined by quantitative real time RT-PCR. The bar graphs show gene expression levels relative to those in Panc1 cells. (B) DNA methylation of the *MUC1 *promoter region examined by MSE in Caco2 cells (high methylation control) and LS174T cells (low methylation control). (C). Expression of MUC1 protein examined by immunohistochemical staining. N: normal tissue. T: tumor tissue. All isolated crypt samples showed high expression levels of MUC1 mRNA and protein, and hypomethylation of the promoter region.

In ethanol-fixed isolated crypts from human colonic normal mucosa and carcinoma, the expression level of mRNA and the DNA methylation status were analyzed. Immunohistochemical staining for MUC1 was performed using serial sections of ethanol-fixed isolated crypts from human colonic normal mucosa and carcinoma lesions. Both normal and neoplastic crypts showed a high expression level of mRNA (Figure 4A) in a real time PCR assay, low methylation of the MUC1 promoter region in MSE analysis (Figure 4B), and positive MUC1 expression in immunohistochemistry (Figure 4C). All isolated crypt samples were found to have a hypomethylated MUC1 promoter by MSE. Similar analysis performed for MUC2 and MUC4 gave similar results (Additional file 6: Figure S3). Thus, these results suggest that the MSE method allows analysis of the extent of DNA methylation in ethanol-fixed sections.

**Figure 5:**
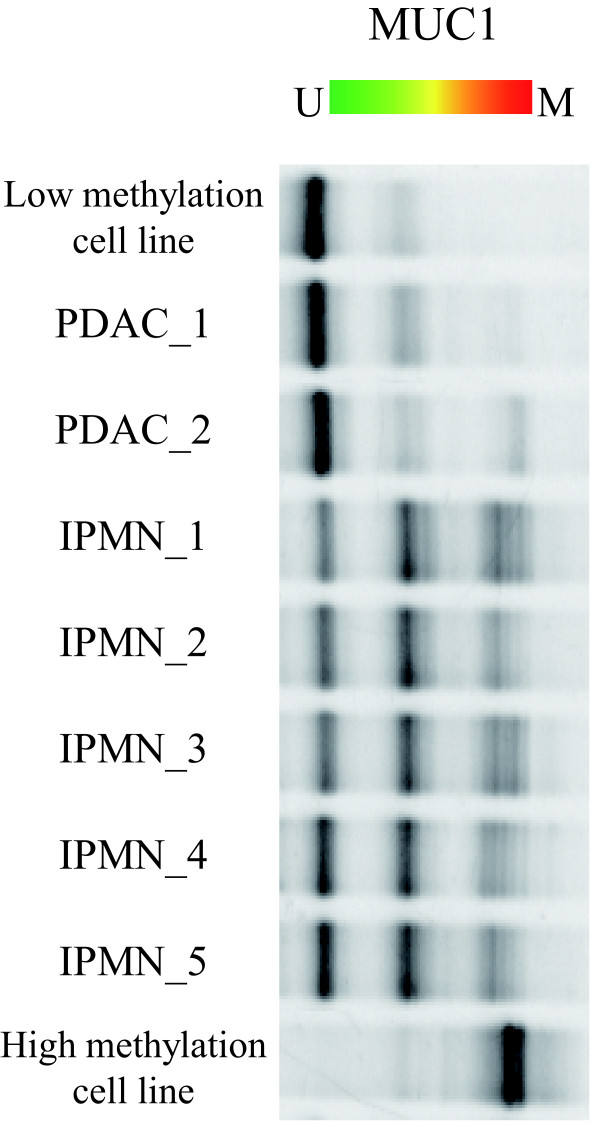
**MSE analysis of MUC1 promoter DNA methylation status using human fluid samples**. Pancreatic juice samples were collected from 2 patients with PDAC and 5 patients with IPMN. The level of methylation in PDAC was significantly lower than that in IPMN.

To examine whether the MSE method can be used to analyze human fluid samples, we applied MSE to analysis of DNA obtained from human pancreatic juice. Pancreatic juice samples were collected from 2 patients with PDAC and 5 patients with IPMN. The amount of recovered DNA was 2.3 μg (PDAC-1), 2.7 μg (PDAC-2), 904 ng (IPMN-1), 2.3 μg (IPMN-2), 132 ng (IPMN-3), 72 ng (IPMN-4), and 100 ng (IPMN-5). Then, bisulfite treated processing was performed to the sample of 1 μg (PDAC-1), 1 μg (PDAC-2), 452 ng (IPMN-1), 1 μg (IPMN-2), 132 ng (IPMN-3), 72 ng (IPMN-4), and 100 ng (IPMN-5), respectively. The 1/20 volume of the bisulfite treated DNA was applied for MSE and conventional bisulfite-DGGE. Although the DNA was recovered in small amounts and/or low quality, the MSE method was sufficiently robust to allow detailed analysis of the DNA methylation patterns in all the samples, and showed significant different patients between PDAC and IPMN (Figure 5). In contrast, the conventional bisulfite-DGGE method could not detect the methylation status in any samples (data not shown). Similar analyses performed for MUC2 and MUC4 gave similar results (Additional file 7: Figure S4). Thus, these results indicate that MSE is applicable for various types of crude samples, including human fluid samples, whereas the conventional bisulfite-DGGE method could not detect the methylation status.

To our knowledge, there is no report describing this intuitive modification of the bisulfite-DGGE method. Nobody could show the diagnostic usefulness of DNA methylation status of mucin genes for pancreatic juices. Interestingly, in the pancreatic juice analysis, the electrophoresis band pattern of MSE revealed a significant difference of degree of methylation status in the promoter region of MUC1, MUC2 or MUC4 between PDAC with a very poor prognosis and IPMN with a favorable prognosis. A further study is needed to clarify the biological significance of this observation, but the lower methylation in PDAC compared to IPMN may provide an approach for early diagnosis of pancreatic neoplasm.

## Conclusions

The MSE method can provide a qualitative information of methylated sequence profile. The MSE method is a simple, sensitive, and specific method for determining the DNA methylation status of almost any CpG island. Here, we have shown that MSE can be used to analyze ethanol-fixed samples (human colonic crypts) and human fluid samples (human pancreatic juice). Thus, MSE can be applied to analysis of DNA methylation in many different clinical samples, and this may facilitate identification of new risk markers.

## Competing interests

The authors declare that they have no competing interests.

## Authors' contributions

SY, SK, NY were carried out the methylation analysis using MSE, SY, SK, NY, IH analyzed the mRNA expression data; TS, SN, YA, KT collected clinical samples and data; MH, SY were carried out the clinicopathologic analysis; SY, SK, SY were involved in the conceptual design of the study; SY was responsible for the supervision of the project, SY and SY wrote the manuscript. All authors read and approved the final manuscript

## Pre-publication history

The pre-publication history for this paper can be accessed here:

http://www.biomedcentral.com/1471-2407/12/67/prepub

## Supplementary Material

Additional file 1 **Table S1**. Synthetic oligonucleotides used in RT-PCR. Click here for file

Additional file 2 **Table S2**. MassARRAY analysis of MUC1 promoter region at Caco2 and T-47D. Click here for file

Additional file 3 **Table S3**. Sequence of MUC1 promoter region, and bisulfite-sequence of *Sss *I treated DNA of Caco2 and PCR amplicon of T-47D. Click here for file

Additional file 4 **Figure S1**. Preparation of fully methylated and unmethylated controls. The methyltransferase (*Sss *I) treatment of 1 μg DNA was performed at 37°C for 4 h. The fully unmethylated control was construct by PCR with the following primers (forward primer 1: 5'-CATTATCCAGCCCTCTTATTTCTC-3' and reverse primer 2: 5'-ACTTCTCTACAGGACATTTGCTTG-3') using 20 ng of DNA as template. Then, these DNA samples were applied to bisulfite treatment. Click here for file

Additional file 5 **Figure S2**. Analysis of the methylation status of DNA extracted from PDAC patient. Five concentration samples were prepared by a ten-fold serial dilution using initial bisulfite treated DNA sample (20 ng/μl). D.W.: using distilled water; N.T.: using non-bisulfite treated DNA. Click here for file

Additional file 6 **Figure S3**. MSE analysis of MUC2 and MUC4 promoter DNA mathylation status using human colonic normal and neoplastic crypts. N:normal tissue. T: tumor tissue. All isolated crypt samples showed high expression levels of MUC2 and MUC4 mRNA and protein. Click here for file

Additional file 7 **Figure S4**. MSE analysis of MUC2 and MUC4 promoter DNA methylation status using human fluid samples. Pancreatic juice samples were collected from 2 patients with PDAC and 5 patients with IPMN. The level of methylation in PDAC was significantly lower than that in IPMN. Click here for file
