# Supplementary material for: The application of methylation specific electrophoresis (MSE) to DNA methylation analysis of the 5' CpG island of mucin in cancer cells
Source: BMC Cancer. 2012 Feb 14;12:67. doi: 10.1186/1471-2407-12-67 (PMC3311064; doi:10.1186/1471-2407-12-67)
Supplement: Additional file 1 — Table S1. Synthetic oligonucleotides used in RT-PCR. [file 1471-2407-12-67-S1.DOC]

| Supplementary Table 1. Synthetic oligonucleotides used in RT-PCR | | |
| --- | --- | --- |
| Primer name | | sequence |
| MUC1 | Forward: | 5’-CCAGCACCGACTACTACCAAGAG-3’ |
|  | Reverse: | 5’-CGTCGTGGACATTGATGGT-3’ |
| MUC2 | Forward: | 5’-ACTTTGATCCTCCCAGACAG-3’ |
|  | Reverse: | 5’-TTGCCCTGGTAGCTGTAGTAG-3’ |
| MUC3A | Forward: | 5’-TCCGGATGGTGGGGCGG-3’ |
|  | Reverse: | 5’-ACACTGAGGACGAGGT-3’ |
| MUC4 | Forward: | 5’-TGGGACGATGCTGACTTCTC-3’ |
|  | Reverse: | 5’-CCCCGTTGTTTGTCATCTTTC-3’ |
| MUC5AC | Forward: | 5’-AAACCTACAACAACATCATCAGG-3’ |
|  | Reverse: | 5’-CTCGTAGTTGAGGCACATCTT-3’ |
| MUC17 | Forward: | 5’-GCTGTGTCTGCTGACCTTGG-3’ |
|  | Reverse: | 5’-TGGCACTGACGGTTCAAGAC-3’ |
| GAPDH | Forward: | 5’-CAACGGATTTGGTCGTATTG -3’ |
|  | Reverse: | 5’-GGACTCCACGACGTACTCAG -3’ |

Yokoyama et al - Supplementary Table 1
