# Supplementary material for: The application of methylation specific electrophoresis (MSE) to DNA methylation analysis of the 5' CpG island of mucin in cancer cells
Source: BMC Cancer. 2012 Feb 14;12:67. doi: 10.1186/1471-2407-12-67 (PMC3311064; doi:10.1186/1471-2407-12-67)
Supplement: Additional file 2 — Table S2. MassARRAY analysis of MUC1 promoter region at Caco2 and T-47D. [file 1471-2407-12-67-S2.DOC]

| Supplementary Table 2. MassARRAY analysis of MUC1 promoter region at Caco2 and T-47D . | | | | | | | | |
| --- | --- | --- | --- | --- | --- | --- | --- | --- |
| CpG site Number | 1 | 2 | 3-5 | 6 | 7 | 8 | 9 | 10 |
| Caco 2 | 92 % | 74 % | 100 % | 85 % | 98 % | 100 % | 100 % | 96 % |
| T-47D | 1 % | 0 % | 3 % | 0 % | 4 % | 3 % | 2 % | 2 % |
| methylated parcentage | | | | | | | | |

Yokoyama et al - Supplementary Table 2
