# Supplementary material for: The application of methylation specific electrophoresis (MSE) to DNA methylation analysis of the 5' CpG island of mucin in cancer cells
Source: BMC Cancer. 2012 Feb 14;12:67. doi: 10.1186/1471-2407-12-67 (PMC3311064; doi:10.1186/1471-2407-12-67)
Supplement: Additional file 3 — Table S3. Sequence of MUC1 promoter region, and bisulfite-sequence of Sss I treated DNA of Caco2 and PCR amplicon of T-47D. [file 1471-2407-12-67-S3.PDF]

Supplementary Table 3. Sequence of MUC1 promoter region, and bisulfite-sequence of SssI treated DNA of Caco2 and PCR amplicom of T-47D.

CpG site No	1 2 3 4 5 6 7 8 9 10
MUC1 promoter region	**gCGgggttttgtcacctgtcacctgctcCGgctgtgcctagggCGggCGggCGgggagtggggggacCGgtataaagCGgtaggCGcctgtgccCGctccacctctcaagcagccagCGc**
bisulfite-treated	
CpG site No	1 2 3 4 5 6 7 8 9 10
*Sss*I-treated Caco2 DNA	**gCGgggttttgttatttgttatttgtttCGttgtgtttagggCGggCGggCGgggagtggggggatCGgtataaagCGgtaggCGtttgtgttCGttttattttttaagtagttagCGt**
bisulfite-treated	
CpG site No	1 2 3 4 5 6 7 8 9 10
T-47D PCR product	**gTGgggttttgttatttgttatttgtttTGttgtgtttagggTGggTGggTGgggagtggggggatTGgtataaagTGgtaggTGtttgtgttTGttttattttttaagtagttagTGt**
