# Supplementary figures and images for: The application of methylation specific electrophoresis (MSE) to DNA methylation analysis of the 5' CpG island of mucin in cancer cells
Source: BMC Cancer. 2012 Feb 14;12:67. doi: 10.1186/1471-2407-12-67 (PMC3311064; doi:10.1186/1471-2407-12-67)

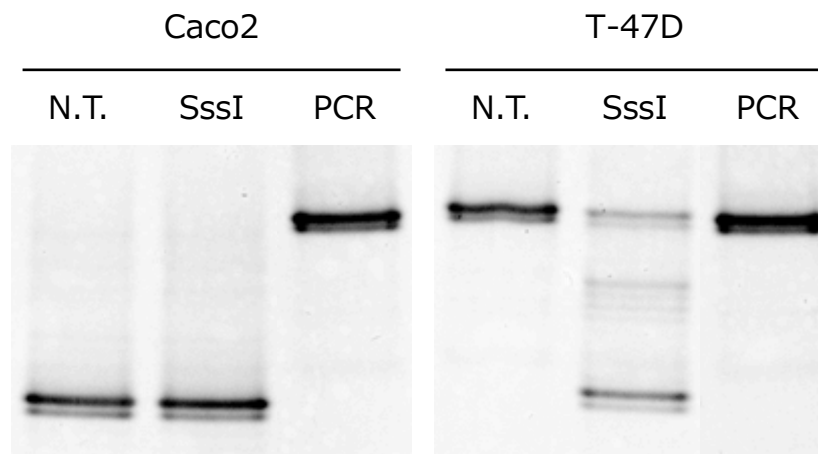

Supplement: Additional file 4 — Figure S1. Preparation of fully methylated and unmethylated controls. The methyltransferase (Sss I) treatment of 1 μg DNA was performed at 37°C for 4 h. The fully unmethylated control was construct by PCR with the following primers (forward primer 1: 5'-CATTATCCAGCCCTCTTATTTCTC-3' and reverse primer 2: 5'-ACTTCTCTACAGGACATTTGCTTG-3') using 20 ng of DNA as template. Then, these DNA samples were applied to bisulfite treatment. [file 1471-2407-12-67-S4.PDF]

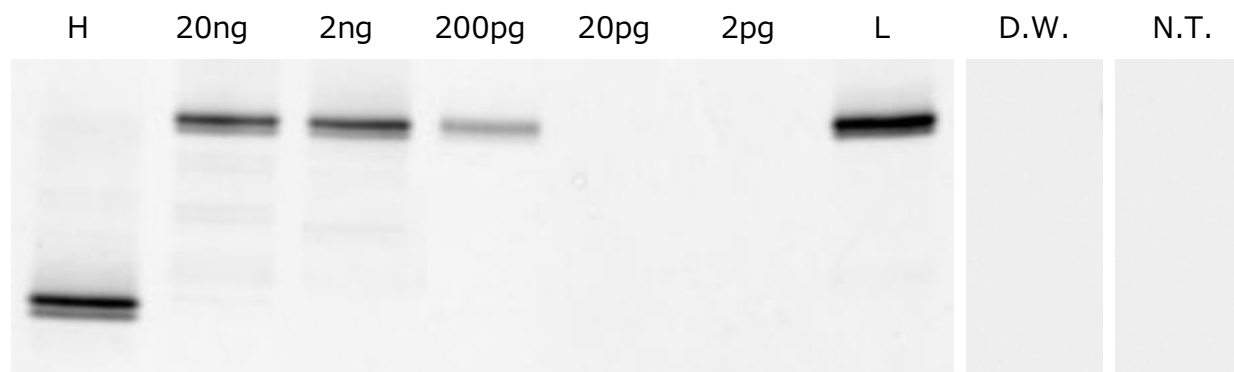

Supplement: Additional file 5 — Figure S2. Analysis of the methylation status of DNA extracted from PDAC patient. Five concentration samples were prepared by a ten-fold serial dilution using initial bisulfite treated DNA sample (20 ng/μl). D.W.: using distilled water; N.T.: using non-bisulfite treated DNA. [file 1471-2407-12-67-S5.PDF]

## MUC2

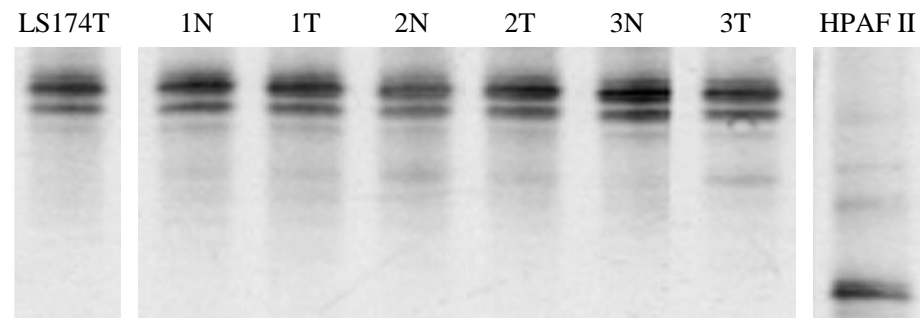

## MUC4

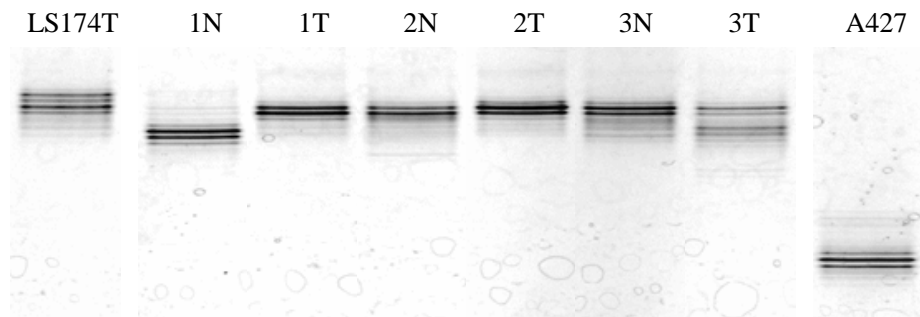

Supplement: Additional file 6 — Figure S3. MSE analysis of MUC2 and MUC4 promoter DNA mathylation status using human colonic normal and neoplastic crypts. N:normal tissue. T: tumor tissue. All isolated crypt samples showed high expression levels of MUC2 and MUC4 mRNA and protein. [file 1471-2407-12-67-S6.PDF]

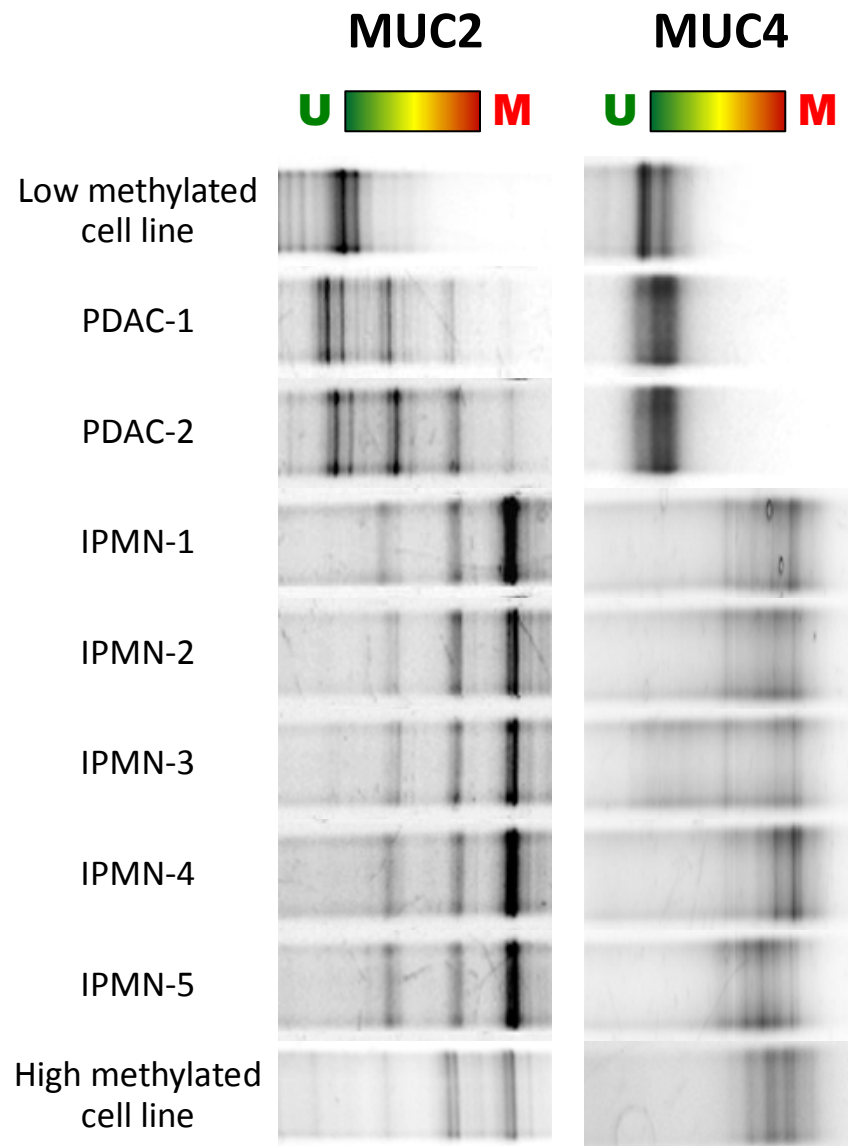

Supplement: Additional file 7 — Figure S4. MSE analysis of MUC2 and MUC4 promoter DNA methylation status using human fluid samples. Pancreatic juice samples were collected from 2 patients with PDAC and 5 patients with IPMN. The level of methylation in PDAC was significantly lower than that in IPMN. [file 1471-2407-12-67-S7.PDF]
